# The cost of a healthy diet and its association with BMI in crisis-stricken Lebanon

**DOI:** 10.1017/S1368980026102092

**Published:** 2026-02-19

**Authors:** Maha Hoteit, Myriam Abboud, Maroun Khattar, Rana Rizk

**Affiliations:** 1 Lebanese University, Lebanon; 2 Zayed University, United Arab Emirates; 3 Lebanese American University, Lebanon

**Keywords:** Health, diet affordability, Mediterranean Diet, food insecurity, nutrition transition, Lebanon

## Abstract

**Objectives:**

This study aimed to determine the prevalence of Lebanese adults living with underweight, overweight or obesity, assess and compare the cost of the Mediterranean Diet (MD) with that of the current dietary consumption pattern and explore diet cost as a determinant of living with underweight, overweight or obesity.

**Design:**

Data for this nationally representative cross-sectional study were collected through sociodemographic questionnaires, anthropometric measurements, the Arab Family Food Security Scale and dietary assessments using a validated FFQ and 24-h recalls. Diet costs were calculated based on 2023 market prices using purchasing power parity. Logistic regression was used to assess associations with BMI.

**Setting:**

Lebanon, using data representative of the Lebanese adults’ population.

**Participants:**

444 Lebanese residents aged 18–64 years.

**Results:**

Overall, 66·2 % of the participants were living with underweight (4·3 %), overweight (37·8 %) or obesity (24·1 %). On average, the cost of following MD ranged from Intl.$ 23·36 to Intl.$ 26·49/person/d, whereas a Lebanese adult spent Intl.$ 20·46 on consumption. Only 31·1 % of participants spent an amount equal to or greater than the minimum MD cost (Intl. $23·36/d). Participants who meet or exceed this threshold were 1·59 times more likely to be living with a healthy weight (aOR = 1·59, *p* = 0·043).

**Conclusions:**

The high prevalence of Lebanese adults living with underweight, overweight or obesity is compounded by the unaffordability of a healthy MD. Improving the affordability of nutritious foods is crucial to promoting healthier dietary patterns and achieving better weight outcomes. Public health strategies should include economic, behavioural and policy-level interventions to enhance diet quality and affordability in crisis-affected populations.

## Introduction

Living with underweight, overweight or obesity negatively impacts individuals and populations worldwide^(1)^. Obesity is a multifactorial disease resulting from genetics, psychosocial factors and obesogenic environments^(2)^. The effects of the latter are through the limited availability of healthy and sustainable food at locally reasonable prices^(2)^. As for underweight, a leading contributing factor is household food insecurity (FI)^(3)^, which limits a household’s ability to access safe, healthy and enough food at all times^(4)^.

The cost of a healthy diet often presents a barrier for healthy eating. In many settings, a healthy dietary pattern is unaffordable with its cost exceeding 30 % of a household’s income^(5)^. According to the World Bank, the average cost of a healthy diet is USD 3·48 per person per day in low-income countries and USD 3·78 in high-income countries^(6)^; this figure exceeds the international poverty line (USD 2·15 per person/d), posing a major challenge for low-income populations^(6)^. Furthermore, the perception of a healthy diet being unaffordable, even when not entirely accurate, can deter individuals from purchasing healthy foods and lead them to opt for cheaper, less nutritious options, thereby contributing to underweight, overweight or obesity^(5)^. In the USA, in children, a higher price of fruits and vegetables was positively associated with lower fibre intake and a higher BMI^(7)^. The specific types of foods available and their relative prices also play a significant role. Due to this, improving the affordability of nutritious foods is a key factor to enhancing population-level dietary behaviours^(5)^.

Among the many healthy diets that exist, the Mediterranean Diet (MD) is considered a sustainable diet given its lower environmental impact due to its emphasis on plant-based foods including fruits, vegetables, whole grains and legumes and moderate consumption of fish, with low consumption of red and processed meat. The MD also supports sustainability through its cultural heritage, local economic benefits and promotion of biodiversity and traditional, seasonal food systems^(8,9)^. As such, encouraging adherence to the MD by making it affordable can improve the health of both the environment and humans.

Worldwide, there is a significant increase in the prevalence of people living with underweight, overweight and obesity. Especially, there is an alarming increase in the prevalence of people living with obesity, whose prevalence increased from 6·6 per cent to 15·8 per cent between the years 1990 and 2022, with 890 million adults affected in 2022 and projections of one billion adults having obesity by 2030^(10)^. Obesity is now prevalent in low- and middle-income countries while historically, it was more common in high-income countries^(10)^. Specifically, the prevalence of adult obesity is surging in the Eastern Mediterranean Region (EMR), increasing from 15·1 per cent in 1980 to 20·7 per cent in 2015^(11)^. This rise is driven by the rapid economic, lifestyle and demographic changes, especially decreased levels of physical activity and the shift in consumption patterns from traditional to Western diets^(11,12)^.

This is the case of Lebanon, a country in the EMR, where the obesity rates are increasing^(13)^, and where the dietary behaviours of the population have shifted from the traditional diet, rich in healthy fats and dietary fibre, towards the Western diet, characterised by increased consumption of ultra-processed foods that are high in Na, trans-fatty acids, saturated fats and added sugars and low in fibre and essential vitamins and minerals^(14,15)^. A recent national consumption study among Lebanese adults revealed that ultra-processed food consumption contributed the most to their total energy intake, reaching 46·7 per cent^(15)^. The concomitant low dietary diversity, low adherence to healthy diets and low dietary intake of phytochemicals and essential vitamins and minerals^(14–17)^ put the Lebanese adults’ population at an increased risk of having malnutrition, Non-Communicable Diseases (NCD) and other complications^(14–17)^. For instance, adults in Lebanon have low adherence to the MD, the EAT-Lancet diet and the US Department of Agriculture (USDA) diet. Their dietary pattern poor in healthy fats and vitamins A, D and E. Specifically, women of reproductive age are not meeting their daily Ca, vitamin D, Fe and vitamin B_12_ requirements^(14,16,17)^. Factors like economic instability, increased food prices and lifestyle changes are contributing to these deficiencies^(14)^. Given Lebanon’s limited progress towards achieving diet-related NCD targets, monitoring trends in living with underweight, overweight or obesity and determining its correlates are of utmost importance.

To date, no previous studies in Lebanon assessed the association between BMI and diet price. This study was conducted, amid an unprecedented economic, health and security crisis the country, to (1) determine the prevalence of Lebanese adults living with underweight, overweight or obesity, (2) assess the cost of following a healthy diet and compare it with the cost of the current consumption pattern and (3) explore diet cost as a determinant of living with underweight, overweight or obesity. By providing new insights into this population’s dietary pattern from an economic perspective, such findings inform tailored interventions and effective preventive measures that would benefit the population’s overall health and well-being.

## Methods

### Study design

A cross-sectional study was conducted between May 2022 and September 2022 on a nationally representative sample (*n* 444) of Lebanese adults aged between 18 and 64 years. To be eligible, participants had to be an adult of Lebanese nationality. The study period was characterised by the economic crisis with depreciation of the national currency, the coronavirus pandemic and the aftermath of the Beirut port explosion.

### Sampling technique

**Figure 1.**
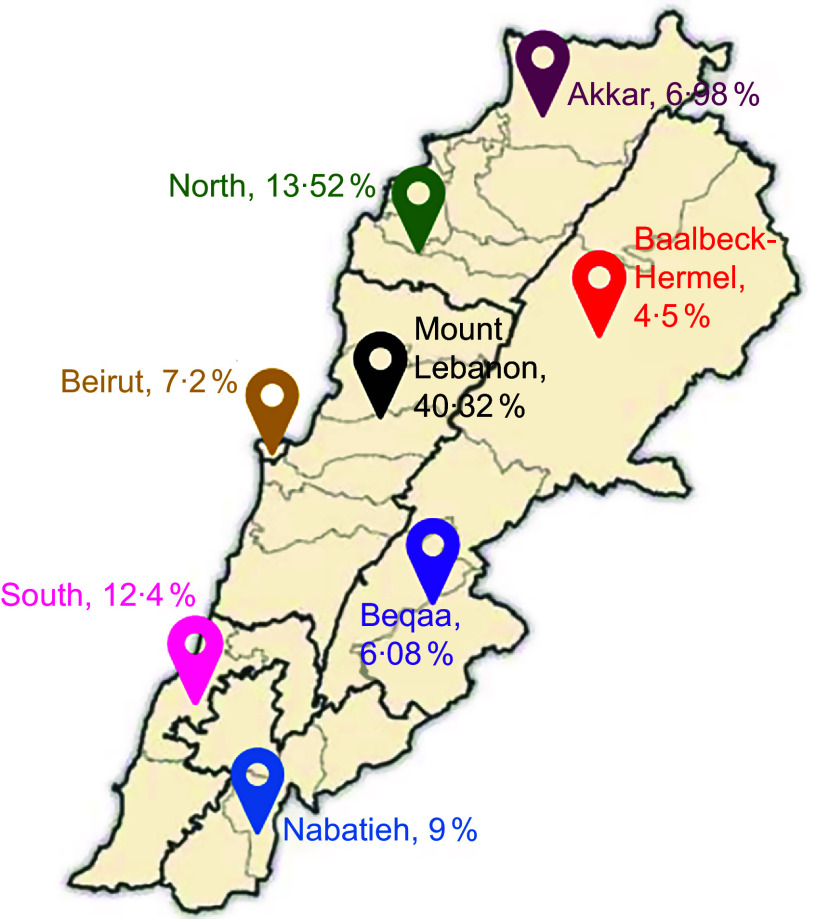
Distribution of study participants^(19)^.

To calculate the sample size, the following formula was applied using population estimates from 2018 to 2019: *n* = [*p*(1 − *p*)] × [(*Z*_∝/2_)^2^/(*e*)^2^]^(18)^. In this formula, ‘*n*’ corresponds to the sample size, ‘*p*’ corresponds to the probability of adults unable of taking precautions against diseases (50 %), ‘*e*’ corresponds to the tolerated level of standard error (5 %) and ‘*Z*_*∝*/2_’ corresponds to the reliability coefficient related to the standard error at a 5 % significance level (1·96)^(18)^. Based on this, 400 participants were needed for the sample to be representative. To compensate for dropouts, a total of 449 participants were included in the study. Of the 449 participants, five were excluded due to errors or missing data. A stratified cluster sampling technique was applied in our study. The eight Lebanese governorates were used as clusters, meaning all adult individuals within each governorate were considered as a group for sampling. Simultaneously, gender was used to create two strata (male and female) within each governorate. This ensured the representation of both genders across all geographic regions. Figure 1 shows the distribution of participants across the different Lebanese governorates.

### Data collection

Face-to-face methods were used for anthropometric measurements, while phone-based interviews were used for food consumption: FFQ, 24-h recalls (24HR) and questionnaires of the Arab family food security scale (AFFSS) and sociodemographic variables.

#### Sociodemographic and health characteristics questionnaire

A pre-tested questionnaire that asked about the study participants’ gender, age, residence, marital status, employment status, educational level and presence of chronic conditions was administered in Arabic to collect sociodemographic and health data. Furthermore, information about the size of the household and the number of rooms was recorded in order to compute the crowding index, which represents the socio-economic standing of the household^(20)^. The calculation of the crowding index involves dividing the number of persons living in a household by the number of rooms in the household (excluding bathrooms and kitchens); a household with one person or fewer per room is deemed not crowded, whereas a household with more than one person per room is deemed crowded^(20)^.

#### Anthropometric measurements

Each participant’s weight and height were measured three times using specific, consistent methods and properly calibrated equipment^(21)^, that is, a digital scale for weight and stadiometer for height. The average of the three measurements was used to calculate the BMI. After computing the BMI, participants were classified as either living with a healthy weight if their corresponding BMI was within the normal range (18·5 and 24·9 kg/m^2^) or living with underweight, overweight or obesity if their corresponding BMI was not within the normal range (below 18·5 or above 24·9 kg/m^2^)^(22)^.

#### Food security status

To assess food security, we used the AFFSS, a tool validated for use in League of Arab States countries like Lebanon. The AFFSS evaluates food security based on household experiences, asking seven questions about access and frequency of food consumption within the past 6 months using a ‘yes’, ‘no’ or ‘do not know’ response format. A question answered with a ‘Yes’ gets a score of 1; otherwise, it gets a score of 0. The overall score is calculated by adding up the points from all ‘Yes’ responses; scores of 0–2 indicate food security, while 3–7 suggest that a household has experienced FI^(23)^.

#### Food consumption

Food consumption of the study participants was collected using a combination of an FFQ and two non-consecutive 24HR, administered on three separate days. The FFQ, composed of 157 food items, was administered in Arabic language and is validated for use in the Lebanese adults’ population^(24)^. It asked about the frequency of consumption as either daily, weekly or monthly. Following the collection of the FFQ data, monthly intake was divided by 30 and weekly consumption by 7 to get daily consumption in grams per day (g/d). As for the 24HR, surveys were conducted during the same study period and participants were asked to list all the meals and beverages they had consumed during the previous 24 h on a typical weekday and one weekend day. Following the collection of the food consumed, the 24HR items were categorised into food groups (vegetables, fruits, breads, dairy products, etc.) based on the FFQ items, and the average intake was calculated using the following formula: [value (FFQ) + [value (24HR)]/2. The information was gathered through 30-min phone interviews during the three separate days with qualified dietitians, who provided participants with visual aids and instructions ahead and during the day of data collection to help them better recall the food consumed and estimate the portion size as accurate as possible.

#### Cost of a healthy diet and consumption pattern

To calculate the cost of a healthy diet, we assessed the price of following the MD recommendations, which is the traditional diet in Lebanon and one of the most popular and healthiest diets globally. The MD encourages the consumption of vegetables, olive oil, fruits and whole grains, while limiting the consumption of red and processed meat and sweets^(25)^. To calculate the MD and dietary pattern cost for an adult individual in the Lebanese population, the recommended amount to be consumed of each food group^(26)^ and the consumed amount (g/d) were multiplied by the average price in Lebanese Pound (LBP) of the corresponding food group in the Lebanese market based on prices set by the ‘Lebanese Ministry of Economy and Trade’ for the year 2023^(27)^. Prices were then converted to International dollar (Intl. $) using purchasing power parity of the year 2023, during which Intl. $ 1 corresponded to LBP 23 736·79^(28)^. Food items were categorised according to the MD classification^(26)^, and the recommended and consumption costs for each food group and in total were computed. Based on the MD recommendations, some categories had a minimum and maximum price (fruits, eggs and grains/cereals), others had an at least price (vegetables, legumes, fish/shellfish and olive oil), others had a maximum price that should be exceeded (red/processed meats, potato and sweets) and others had an exact price (white meat, milk/dairy products and olive/nuts/seeds). Food items included in each food group are shown in Supplementary Table S1.

### Data management and statistical analysis

Data were managed on Excel and then transferred to the ‘Statistical Package for the Social Sciences, the IBM SPSS Statistics 21’ for analysis. Descriptive statistics were used to summarise quantitative variables, using means and standard deviations for continuous variables and percentages and frequencies for categorical variables. BMI was dichotomised into participants who have a healthy one and those who do not. Chi-square test was used to examine the associations between the various categorical variables and BMI, while Pearson’s correlation was employed to examine the relationship between the continuous variable (total diet cost) and BMI. To explore diet price as a determinant of the dependent variable (BMI), an Enter logistic regression model was used, adjusting for potential confounding factors derived from relevant literature and the bivariate analysis and after checking for collinearity between variables. No problems of collinearity were shown between the studied variables (|*r*| < 0·7). A *P*-value of less than 0·05 was considered as significant, and values were presented as adjusted OR (aOR) at 95 % CI. Supplementary Table S2 displays the findings of an exploratory bivariate analysis that was used to choose the variables that were added to the model.

## Results

### Characteristics of the study participants

Table 1 shows the characteristics of the study participants: 58·8 % females, mean age: 34·1 ± 12·7; 47·5 % residing in Beirut and Mount Lebanon, 62·84 % living in a crowded household, 50·9 % employed, 59·91 % having a university level of education, and 47·3 % having FI. Two-thirds of the sample (66·2 % in the total sample, 42·9 % in males and 57·1 % in females) were living with underweight (4·3 %), overweight (37·8 %) or obesity (24·1 %).

**Table 1.** Sociodemographic and socio-economic characteristics, food security and BMI statuses

		Overall (n 444)		Male (n 183)		Female (n 261)	
		*n*	%	*n*	%	*n*	%
Age category	18 years	20	4·5	5	25	15	75
	19–30 years	187	42·1	78	41·7	109	58·3
	31–50 years	174	39·2	79	45·4	95	54·6
	51–64 years	63	14·2	21	33·3	42	66·7
Residency	Beirut and Mount Lebanon	211	47·5	83	39·3	128	60·7
	North and Akkar	91	20·5	44	48·3	47	51·7
	South and Nabatiyeh	95	21·4	41	43·1	54	56·9
	Bekaa and Baalbek-Hermel	47	10·6	15	31·9	32	68·1
Marital status	Not married	221	49·7	86	38·9	135	61·1
	Married	223	50·3	97	43·5	126	56·5
Crowding index	No crowding	165	37·16	79	47·9	86	52·1
	Crowding	279	62·84	104	37·3	175	62·7
Number of children	0	217	48·87	87	40·1	130	59·9
	1–3	159	35·81	74	46·5	85	53·5
	>3	68	15·32	22	32·4	46	67·6
Educational level	Illiterate to school	178	40·09	73	41	105	59
	University	266	59•91	110	41·4	156	58·6
Employment status	Unemployed	218	49·1	40	18·3	178	81·7
	Employed	226	50·9	143	63·3	83	36·7
Monthly salary changes after economic crisis	No impact	129	29	63	48·8	66	51·2
	Decline in salary	123	27·7	51	41·5	72	58·5
	Increase in salary	70	15·8	42	60	28	40
	Already have no salary	122	27·5	27	22·1	95	77·9
Household monthly income	≤300 USD	400	90·1	152	38	248	62
	>300 USD	44	9·9	31	70·5	13	29·5
AFFSS	Food security	234	52·7	102	43·6	132	56·4
	Food insecurity	210	47·3	81	38·6	129	61·4
BMI	Living with a healthy weight	150	33·8	57	38	93	62
	Living with underweight, overweight or obesity	294	66·2	126	42·9	168	57·1

AFFSS, Arab Family Food Security Scale; USD, United States Dollar.

### Adherence to the Mediterranean Diet among Lebanese adults and price of following the Mediterranean Diet

**Figure 2.**
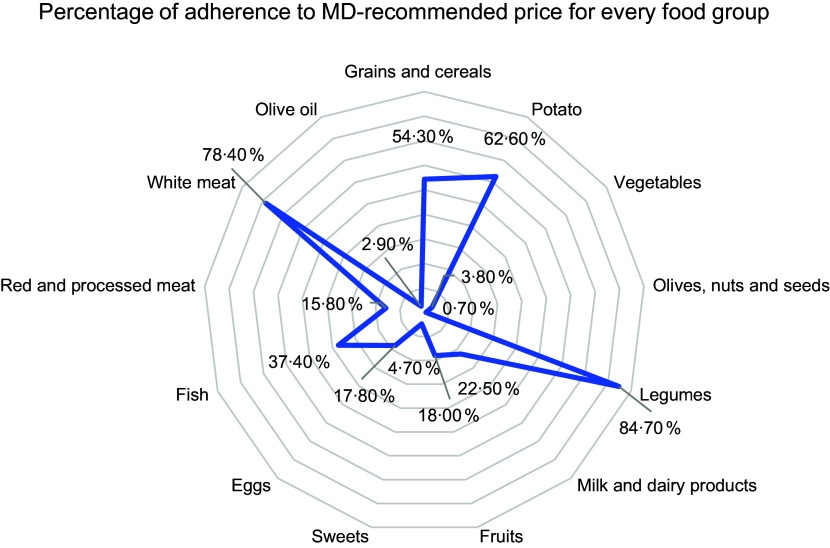
Adherence to the MD-recommended price for every food group. MD, Mediterranean Diet.

Figure 2 details the adherence patterns to the MD among Lebanese adults. A good adherence was shown for legumes (84·7 %) and white meat (78·4 %), while a low adherence was shown for olives/nuts/seeds (0·7 %), olive oil (2·9 %), vegetables (3·8 %) and sweets (4·7 %).

Table 2 shows the minimum and maximum costs of following the MD based on the Lebanese market prices for the year 2023. On average, the cost of following MD ranged from Intl. $ 23·36 to Intl. $ 26·49 per person per d, when the least recommended amounts of vegetables, fish/shellfish, legumes and olive oil are taken. Based on the MD recommendations, milk and dairy products had the highest price (Intl. $ 9·98), followed by olives/nuts/seeds (Intl. $ 8·03).

**Table 2.** Cost of a healthy diet for a Lebanese adult based on the MD recommendations

Food group	Minimum (LBP)	Maximum (LBP)	Minimum (Intl. $)	Maximum (Intl. $)
Fruits (g/d)	25 944·3	51 888·6	1·09	2·19
Vegetables (g/d)	At least 40 742		At least 1·72	
Eggs (g/d)	4173·55	8347·11	0·18	0·35
White meat (g/d)	3465·29	3465·29	0·15	0·15
Potato (g/d)	0	1813·11	0·00	0·08
Legumes (g/d)	At least 4222·13		At least 0·18	
Milk and dairy products (g/d)	236 821	236 821	9·98	9·98
Olive oil (ml/d)	At least 22 198		At least 0·94	
Olives, nuts and seeds (g/d)	190 544	190 544	8·03	8·03
Grains and cereals (g/d)	19 939·92	39 879·84	0·84	1·68
Fish and shellfish (g/d)	At least 6462		At least 0·27	
Sweets (g/d)	0	11 071	0·00	0·47
Red and processed meat (g/d)	0	11 423	0·00	0·48
**Total**	**554 512·88**	628 877·75*	**23·36**	26·49*

MD, Mediterranean Diet; LBP, Lebanese Pound; Intl. $, International Dollar.

Prices reported are per person per day for the year 2023.

*Maximum price if the minimum recommended amount of vegetables, fish/shellfish, legumes and olive oil is taken.

Table 3 shows the cost of the dietary pattern followed by the Lebanese adults’ population. On average, a Lebanese adult spent Intl. $ 20·46 on consumption, mostly corresponding to milk and dairy products (Intl. $ 6·87), followed by sweets (Intl. $ 4·92). On average, 31·1 % of the population had a consumption cost equal to or higher than the minimum cost of following the MD.

**Table 3.** Cost of the dietary pattern followed by a Lebanese adult (per person per day)

Food group	Average price (LBP)	Average price (Intl. $)
Grains and cereals	36 995·07	1·56
Potato	1819·27	0·08
Vegetables	19 441·13	0·82
Fruits	21 148·5	0·89
Legumes	11 198·49	0·47
Nuts and seeds	30 226·58	1·27
Olive oil	6492·63	0·27
Milk and dairy products	163 029·8	6·87
Eggs	6511·93	0·27
Fish	9738·64	0·41
White meat	15 225·14	0·64
Red and processed meat	47 154·3	1·99
Sweets	116 755·58	4·92
Total	485 737·06	20·46
Percent of population whose consumption expenditure is equal to or above the minimum MD cost (Intl. $ 23·36)	*n*	%
	138	31·1 %

LBP, Lebanese Pound; Intl. $, International Dollar; MD, Mediterranean Diet.

### Diet cost as a determinant of healthy BMI

Variables correlated with a BMI are shown in Supplementary Table S2. These variables were entered in the logistic regression model whose results are shown in Table 4. Participants having a consumption expenditure equal to or more than the minimum MD recommendation (23·36 Intl. $) were 1·59 times more likely to have a healthy BMI (aOR: 1·59, *P*-value = 0·043).

**Table 4.** Factors associated with healthy BMI

	Healthy BMI			
	aOR	95 % CI		*P*-value
		Lower	Upper	
Age category				
18 years (reference)	–	–	–	–
19–30 years	1·64	0·61	4·39	0·33
31–50 years	0·92	0·33	2·54	0·87
51–64 years	0·37	0·10	1·30	0·12
Educational level				
Illiterate to school (reference)	–	–	–	–
University	**2·43**	**1·45**	**4·09**	**0·001**
AFFSS				
Food secure (reference)	–	–	–	–
Food insecure	0·97	0·62	1·52	0·88
Consumption expenditure				
< 23·36 Intl. $ (Reference)	–	–	–	–
≥ 23·36 Intl. $	**1·59**	**1·001**	**1·13**	**0·043**

aOR, adjusted OR; AFFSS, Arab Family Food Security Scale; Intl. $, International dollar.

*P*-value in bold is significant. *23.36 Intl. $ is the minimum recommended MD price per person per day*. Variables entered in the model: age category, AFFSS, education level and consumption expenditure.

## Discussion

This study assessed the prevalence of living with underweight, overweight or obesity on a nationally representative sample of Lebanese adults amid a triple crisis. In addition, it is first to assess the cost of following the traditional healthy diet, that is, the MD, and compare it with the cost of the current consumption pattern, and the first to explore diet cost as a determinant of healthy BMI. As such, this study pioneers in giving an economic perspective into the dietary behaviours of the Lebanese adult population that is experiencing a shift towards the Westernised diet^(14,15)^. The current findings revealed that almost two-third of Lebanese adults live with underweight, overweight or obesity (66·2 %), with 4·3 % living with underweight, 37·8 % living with overweight and 24·1 % living with obesity. These figures remain higher than the regional average of 20·7 %^(11)^ and the global average of 16 %^(2)^, warranting continued mitigation strategies.

Only 31·1 % of our study participants had a consumption expenditure higher than the minimum price of MD, suggesting that the majority of the population is unable to afford a healthy diet. In addition, participants who had a consumption expenditure equal to or more than the minimum recommended MD were more likely to have a healthy BMI: the cost of a diet could hence influence weight by affecting food choices and dietary patterns^(29)^. Generally, healthier foods tend to be more expensive per calorie than less healthy, processed options, and individuals with lower income could be led to choose less expensive, calorie-dense foods, potentially contributing to weight gain^(30)^. Plus, participants who have a higher consumption expenditure are more likely to be able to afford a diversified and high-quality diet^(31)^, which is associated with a healthier BMI. Since we adjusted our model for FI profile, the current finding indicates that the consumption expenditure is a standalone determinant of BMI, highlighting the important role of diet affordability when it comes to healthy eating and improving BMI.

In addition to consumption expenditure, participants with a university-level education were more likely to have a healthy BMI. Similar findings were found in Europe, where education level was inversely associated with BMI^(32)^. In addition, Iranian adults with lower education levels were shown to have a higher prevalence of obesity, aligning with our findings^(33)^. Potentially, individuals with higher educational attainment are more knowledgeable about the importance of nutrition and healthy eating, leading them to adopt better food choices, such as consuming more fruits and vegetables, and engage in physical activity than their counterparts^(34)^, eventually leading to healthier weight.

The current findings highlight the importance of diet affordability when it comes to public health. Affordability is central to ensuring widespread access to nutritious food, affecting dietary behaviours and health outcomes^(29)^: when healthy diets are affordable, individuals can better meet their dietary needs, which helps in reducing the risk of chronic diseases and improving overall well-being^(35)^. In this context, governments and public health organisations play a key role. For instance, implementing a combination of policies and interventions focused on both the supply and demand sides of the food system, such as fiscal policies including taxes on unhealthy foods and subsidies on healthy options^(36,37)^, institutional and infrastructure reforms to decrease food prices^(38)^, and interventions to improve food access and affordability for vulnerable populations, are some suggested means to enhance affordability. In fact, making food affordable through the distribution of healthy food, or the provision of vouchers for vulnerable populations to purchase healthy food such as fruits and vegetables, led to improved diet quality, reduced FI and allowed better management of chronic diseases in healthcare settings^(39)^. Based on our findings, enhancing the affordability of olive oil, vegetables and olives/nuts/seeds could enhance their consumption, ultimately their health; this requires targeted policies and interventions, especially due to the higher price of these commodities compared with unhealthy options. Specifically, the MD can serve as a sustainable and affordable diet for the Lebanese population. By emphasising plant-based proteins, whole grains and seasonal produce, this diet can improve the health of the Lebanese population without compromising the environmental assets^(40,41)^. In addition, the MD can play an important role in improving the food security status in the country since Lebanon’s domestic production of fruits, vegetables and legumes contributes significantly to its food security. As such, government policies aiming to support farmers through direct payments, subsidies and market price controls, while also aiming to lower consumer costs through measures like food subsidies and rural development programmes^(42)^, are crucial. To balance these goals, the Lebanese government can use a combination of tools such as import quotas and production limits, along with rural development initiatives and assistance with sustainable practices to stabilise markets and reduce costs.

While affordability is a crucial factor in promoting adherence to the MD, interventions focused on behavioural change, education and cultural adaptation are also essential for long-term results^(43)^. To promote sustained adoption, it may be necessary to comprehend and address the psychological, social and environmental variables driving dietary choices^(44)^; merely making the MD more accessible may not be sufficient. For instance, in countries of the Gulf with economic stability, and where affordability is not a problem, a low adherence to the MD was observed, highlighting the complexity of promoting a sustained adoption^(45)^.

### Strengths and limitations

This is the first nationally representative study to assess the cost of a healthy diet and its association with healthy BMI among Lebanese adults. This study also provides updated figures on the prevalence of obesity using a representative sample. In addition, the study uses the combination of FFQ and 24HR to report dietary intake, which increases the accuracy of the results^(46)^. However, this study has some limitations. Data collection relied on self-reported dietary data which are subject to recall bias and may not accurately reflect actual food intake. Additionally, we used data collected in 2022 and applied prices reported in 2023; assuming that consumption did not change. Moreover, we relied on officially published prices, whereas we had at the time black market. Plus, due to the economic crisis, food parcels, cash assistance and food baskets were distributed in the country, which might affect the consumption patterns of the population. These limitations can introduce bias and affect the validity of the findings.

### Conclusions

This study reports a significantly high prevalence of Lebanese adults living with underweight, overweight or obesity, and a consumption expenditure below the minimum recommended MD cost, highlighting unaffordability of our traditional diet. Of interest, this study identifies a consumption expenditure in accordance with the MD recommendations as determinant of a healthy BMI. Efficient policies and interventions enhancing affordability of healthy food items, especially olive oil, nuts/seeds and vegetables, in this population could mitigate living with either underweight, overweight or obesity, eventually improving the health of the population. Future studies should focus on monitoring dietary behaviour changes in this population, as well as changes to the cost and affordability of healthy diets, while also evaluating the effectiveness of relevant policies and interventions.

## Supporting information

Hoteit et al. supplementary material Hoteit et al. supplementary material

## Supplementary material

To view supplementary material for this article, please visit https://doi.org/10.1017/S1368980026102092
