# Supplementary material for: The cost of a healthy diet and its association with BMI in crisis-stricken Lebanon
Source: Public Health Nutr. 2026 Feb 19;29(1):e42. doi: 10.1017/S1368980026102092 (PMC12979016; doi:10.1017/S1368980026102092)
Supplement: Hoteit et al. supplementary material [file S1368980026102092sup001.docx]

**Table S1.** Food items included in each food group

| **Food Groups** | **Items** |
| --- | --- |
| Grains, Cereals and Cereal-Based Products | All kinds of breads, Cereals, rice, rice-based dishes, pasta, oat, bulgur, Lebanese kaake, quinoa |
| Legumes | All kinds of legumes, legume-based dishes, peas and beans, lentils |
| Potato | Potatoes (including potato-based dishes) |
| Vegetables | Raw vegetables (including all kinds of vegetables and salads), cooked vegetables, pickled vegetables, vegetable-based traditional dishes, tomato paste, vegetables soups |
| Olives, Nuts & Seeds | Olives, all kinds of nuts and seeds (including seeds, almonds, cashew nuts, pistachios, walnuts, pine nut, hazelnut) |
| Milk and Dairy Products | All kinds of milk (whole, low fat…) |
|  | All kinds of cheese, yogurt and yogurt-based dishes, laban, kariche, labneh (strained yogurt) |
|  | Milk-based dishes, puddings, frozen and fruit yogurt |
| Red and Processed Meat | Meat and organ meats, processed meat (hotdog, salami, mortadella, sausages) |
| White Meat | Poultry and poultry organs (chickens, birds) |
| Fish | All kinds of seafood (tuna, sardines, fish, seafood) |
| Eggs | Eggs |
| Fruits | All fruits, fruit salads and dried fruits, juices made from 100% fruits |
| Sweets | Candies, cakes, pastries, traditional sweets (jello, custard, Arabic sweets), ice cream, doughnuts, chocolate, biscuits with cream, chips, popcorn, salty biscuits, added sugars, jams, honey, commercial and sweetened juices, soft drinks, energy drinks |
| Olive Oil | Olive oil |

**Table S2.** Factors correlated with BMI

|  | | **BMI** | | | | **P-value** |
| --- | --- | --- | --- | --- | --- | --- |
|  |  | **Living with underweight, overweight, or obesity** | | **Living with a healthy weight** | |  |
|  |  | **N** | **%** | **N** | **%** |  |
| **Age Category** | 18 Years | 13 | 4.4% | 7 | 4.7% | **<0.001** |
|  | 19-30 Years | 97 | 32.9% | 90 | 60.4% |  |
|  | 31-50 Years | 129 | 43.7% | 45 | 30.2% |  |
|  | 51-64 Years | 56 | 19.0% | 7 | 4.7% |  |
| **Gender** | Male | 126 | 42.7% | 57 | 38.3% | 0.368 |
|  | Female | 169 | 57.3% | 92 | 61.7% |  |
| **Marital Status** | Not Married | 145 | 49.2% | 76 | 51.0% | 0.712 |
|  | Married | 150 | 50.8% | 73 | 49.0% |  |
| **Crowding Index** | No crowding | 106 | 35.9% | 59 | 39.6% | 0.450 |
|  | Crowding | 189 | 64.1% | 90 | 60.4% |  |
| **Employment Status** | Unemployed | 146 | 49.5% | 72 | 48.3% | 0.816 |
|  | Employed | 149 | 50.5% | 77 | 51.7% |  |
| **Education level** | Illiterate to school | 147 | 49.8% | 31 | 20.8% | **<0.001** |
|  | University | 148 | 50.2% | 118 | 79.2% |  |
| **AFFSS** | Food secure | 143 | 48.5% | 91 | 61.1% | **0.012** |
|  | Food insecure | 152 | 51.5% | 58 | 38.9% |  |
| **Residency** | Beirut & Mount Lebanon | 134 | 45.4% | 77 | 51.7% | 0.189 |
|  | North & Akkar | 68 | 23.1% | 23 | 15.4% |  |
|  | South & Nabatiyeh | 65 | 22.0% | 30 | 20.1% |  |
|  | Bekaa & Baalbek-Hermel | 28 | 9.5% | 19 | 12.8% |  |
| **Income** | <= 300 USD | 266 | 90.2% | 134 | 89.9% | 0.937 |
|  | > 300 USD | 29 | 9.8% | 15 | 10.1% |  |
| **Consumption Expenditure** | < Intl. $ 23.36 | 216 | 73.2% | 90 | 60.4% | **0.006** |
|  | ≥ Intl. $ 23.36 | 79 | 26.8% | 59 | 39.6% |  |

**Abbreviations:** ***AFFSS*** Arab Family Food Security Scale, ***BMI*** Body Mass Index, ***Intl. $*** International dollar.
